# Type 1 Diabetes and Its Multi-Factorial Pathogenesis: The Putative Role of NK Cells

**DOI:** 10.3390/ijms19030794

**Published:** 2018-03-10

**Authors:** Valeria La Marca, Elena Gianchecchi, Alessandra Fierabracci

**Affiliations:** 1 Type 1 Diabetes Centre, Infectivology and Clinical Trials Research Department, Children’s Hospital Bambino Gesù, Viale San Paolo 15, 00146 Rome, Italy; valeria.lamarca@opbg.net (V.L.M.); elegianche@yahoo.it (E.G.); 2 VisMederi srl, Strada del Petriccio e Belriguardo, 35, 53100 Siena, Italy

**Keywords:** Type 1 Diabetes, NK cells, autoimmune diseases

## Abstract

Type 1 diabetes (T1D) affects millions of people worldwide and is the prevalent form of all pediatric diabetes diagnoses. T1D is recognized to have an autoimmune etiology, since failure in specific self-tolerance mechanisms triggers immune reactions towards self-antigens and causes disease onset. Among all the different immunocytes involved in T1D etiopathogenesis, a relevant role of natural killer cells (NKs) is currently emerging. NKs represent the interface between innate and adaptive immunity; they intervene in the defense against infections and present, at the same time, typical features of the adaptive immune cells, such as expansion and generation of memory cells. Several recent studies, performed both in animal models and in human diabetic patients, revealed aberrations in NK cell frequency and functionality in the peripheral blood and in damaged tissues, suggesting their possible redirection towards affected tissues. NKs oscillate from a quiescent to an activated state through a delicate balance of activating and inhibitory signals transduced via surface receptors. Further accurate investigations are needed to elucidate the exact role of NKs in T1D, in order to develop novel immune-based therapies able to reduce the disease risk or delay its onset.

## 1. Introduction

Diabetes mellitus (DM) is a chronic metabolic disorder caused by either impaired insulin secretion or insulin action, or both [1]. The three broad categories of DM are titled as Type 1 (T1D), Type 2 diabetes (T2D) and gestational diabetes mellitus (GDM) [2]. In T1D, the body’s immune system destroys insulin-releasing β cells, thus cells cannot absorb glucose [2]. Under physiological conditions, β cells work as glucose sensors and regulators, releasing insulin in order to maintain glucose levels within a relatively narrow range [3]. Conversely, in T2D (formerly called adult-onset or non-insulin-dependent diabetes) the body is not able to use insulin appropriately; this is referred to as the phenomenon of insulin resistance. As T2D worsens, the pancreas may progressively produce less insulin, leading to insulin deficiency. Although the pediatric-onset of T2D is becoming more common, T1D continues to be the prevalent form in children, accounting for ~90% of all pediatric diabetes diagnoses [4,5]. GDM refers to glucose intolerance that may occur with onset or first recognition during pregnancy [2]. Furthermore, diabetes can result from monogenic defects in β cell function, diseases of the exocrine pancreas, endocrinopathies due to hormones antagonizing insulin, drug or chemically induced. Uncommon forms of immune-mediated diseases and other genetic syndromes may present with diabetes [2]. 

T1D, affecting millions of people worldwide, represents one of more than 80 diseases recognized to have an autoimmune etiology [3]. Generally, in normal conditions, self-tolerance mechanisms, namely central tolerance [6], prevent intra-thymic maturation and activation of lymphocytes autoreactive to T1D related auto-antigens [7]. Thus, a small fraction of autoreactive cells, escapes from this process, undergoes maturation and reaches the peripheral circulation. To prevent autologous cells destruction by these cells, mechanisms of peripheral tolerance occur destroying or controlling the same autoreactive cells. The failure in central or peripheral self-tolerance mechanisms is responsible for immune reaction to self-antigens and may lead to autoimmune diseases.

T1D is recognized to have a multi-factorial pathogenesis, in which both genetic and environmental factors play important roles [8]. Today, >60 loci associated with T1D have been identified with human leukocyte antigen (HLA) association being the strongest [9]. After HLA, the polymorphism in the promoter region of the insulin gene has the most relevant association (reviewed (rev) in [9]). Only two other loci, protein tyrosine phosphatase non-receptor type 22 (*PTPN22*) and interleukin 2-receptor alpha (*IL-2RA*) have been consistently reported underlying the importance of the HLA region compared with other loci. During the past decade, genome wide association studies (GWAS) improved the identification of risk genes for T1D; notably, 64 single nucleotide polymorphism (SNP)-trait associations for T1D are reported. Nevertheless, the disease-causing variants and genes are still to be largely unraveled [9].

The phenomenon of immune-mediated islet β cell destruction results in lifelong dependence on exogenous insulin and increased risk for the development of secondary complications that affect life quality and duration [10].

### T1D Immunopathology: Looking Inside the Puzzle

From a pathogenic point of view, the disease is the result of a breakdown in immune regulation, i.e., dysregulated thymic and peripheral events that subsequently lead to inflammation of the pancreatic islets typically marked by adaptive and innate infiltrating effectors [3,11]. The natural history of disease shows that the autoimmune process is triggered even years prior to clinical diagnosis [12]. “Insulitis” progresses over time until a sufficient β cell mass is destroyed and/or made nonfunctional. At this point blood glucose levels increase and clinical disease is achieved [3]. Since its first description by Gepts (1965) more than 50 years ago [13] pathologic features have been limited by the scarse access to T1D pancreata.

Various infiltrating cell types are identified in the insulitis lesion and suggest that heterogeneous profiles may underlie disease severity and progression [14]. T and B lymphocytes are present and cytotoxic CD8^+^ T cells are the predominant population that could target β cells expressing high levels of HLA class I molecules (rev in [14]). Interestingly hyper-expression of class I and II molecules support the putative association with viral infections with a role in T1D etiopathogenesis (rev in [14]).

Definitively autoreactive T cells that escaped mechanisms of self-tolerance are the major players in β cell destruction. Several β cell autoantigens are recognized by the islet infiltrating T cells; these include glutamic acid decarboxylase isoform 65 (GAD65), proinsulin, insulin B chain, tyrosine phosphatase-like insulinoma-associated antigen (IA2) and islet-specific glucose-6—phosphatase catalytic subunit-related protein (IGRP) [7]. The loss of immunological self-tolerance is highlighted by the autoimmune process directed towards the islet cell self-antigens. The HLA molecules found to predispose to T1D could be responsible of a suboptimal thymic presentation of autoantigens to the T cell receptor (TCR). This could promote T cell survival to thymic selection. Indeed, increased levels of autoreactive T cells specific for β cell proteins have been depicted from the peripheral blood of newly diagnosed T1D individuals [15].

In the autoimmune process, T helper (Th) cells provide help to autoreactive B cells producing autoantibodies [16]. Once activated/expanded, mature B cells differentiate in plasma cells producing autoantibodies, which, in turn, further contribute to the tissue inflammatory process and destruction. As regards two distinct patterns of insulitis are reported in autopsy samples from newly diagnosed UK patients distinguished by the prevalence of CD20^+^ B cells [17]. A higher proportion of B cells within insulitis is indicative of an earlier autoimmune trigger or more rapid β cell decline. 

By encountering the self- or cross-reactive antigen, Th cells activate, expand and differentiate into various effector subsets, including Th1, Th2 and T regulatory (Treg) cells (rev in [18]). The majority of CD4^+^ and CD8^+^ islet-infiltrating T cells exhibit a Th1 phenotype. Th1 and Th2 cells produce mutually inhibitory cytokine profiles: Th1 cells secrete proinflammatory interleukin 2 (IL-2), IL-1, tumor necrosis factor α (TNF-α) and interferon *γ* (IFN-γ), while Th2 cells secrete controregulatory IL-4, IL-5 and IL-10. A novel distinct CD4+ T cell population, namely Th17, producing IL-17 of still undefined pathogenetic significance was seen in the islets of NOD mice and on pancreatic lymph nodes of T1D patients [19,20]. Forkhead box P3 (Foxp3) Treg play an essential role in regulating immune homeostasis by suppressing T and other effector cells through cell contact and anti-inflammatory mediators [21,22]. Nowadays, B regulatory cells [23] are also recognized as a distinct entity. They express CD5, a well-established negative regulator of TCR [24] and B cell receptor (BCR) signaling [25]. 

Experimental studies also highlight the role of resident islet macrophages in diabetogenesis for their interaction with β cells and blood components [26]. They play distinct functions both contributing to the development and progression of disease by presenting autoantigens to naïve T cells in the draining lymph nodes and as effector cells once islet inflammation is established [27]. They elicit diabetogenic effects by generating nitric oxide (NO) and by producing inflammatory cytokines such as IL-1β and TNF. Dendritic cells (DCs) are the major antigen-presenting cells (APCs) outside and within islets; they play a pivotal regulatory role in T cell immunity, by altering the balance between inflammatory T cells and Treg [28]. The expansion of IFN-α–producing plasmacytoid DCs (pDCs) has been indeed documented in patients with T1D around the time of diagnosis [29].

Yet, several studies have shown cytolytic activity of NK cells against pancreatic islet β-cells and their involvement in the disease development. Indeed, an altered NK cell number and function was found both in the peripheral blood and affected tissues of patients with autoimmune conditions, assuming a possible homing of NKs to the damaged tissues [30]. Depending on the autoimmune disease, NKs show a dual behavior, promoting target cell destruction or protecting against the onset of the autoimmune condition through either positive and negative regulatory effects (rev in [30,31,32,33]).

In this review, we analyze the existing literature on the biology and the putative role of NK cells in the onset and development of T1D as a bridge between innate and adaptive immunity [34]. We also present perspectives derived from our recent insights that open pathways for future research and translational applications.

## 2. Biology of NKs

NK cells are innate lymphocytes activated upon encounter with infected, allogeneic or transformed cells [35,36,37,38,39,40]. However, they also show typical characteristics of the adaptive immune system, such as the expansion of pathogen-specific cells, the generation of long-lasting “memory” cells able to persist upon antigen encounter, and the possibility to induce an increased secondary recall response to re-challenge (rev in [30]). 

NKs, granular and large bone marrow-derived lymphocytes, constitute the third in lineage among lymphocytes, after T and B cells. These cells are classically identified as CD56^+^CD3^−^ cells, distinct from CD56^+^CD3^+^ cells representing a mixed population of NK-like T (NKT) and antigen-experienced T cells showing the up-regulation of several NK cell markers. Based on CD56 levels of expression, NK cells can be distinguished in CD56^dim^ and CD56^bright^ subsets [41]. CD56^dim^ accounts for about 90% of the total NKs in peripheral blood, and it is a mature subpopulation with a high killer cell immunoglobulin-like receptor (KIR) expression; moreover, they are deeply involved in cytotoxicity responses and synthesize little amounts of IFN-*γ*. CD56^bright^ NK cells are characterized by low or no expression of KIRs and elevated IFN-*γ* production. They are more immature and are mostly involved in cytokine production, with a limited role in cytolytic responses. CD56^bright^ subset easily leaves blood vessels and reaches lymph nodes, allowing to hypothesize a process for human NK differentiation that progresses from a CD56^bright^ to a CD56^dim^ phenotype [42]. 

NK cells continuously generate from hematopoietic stem cells (HSC) committed towards NK-cell lineage [43]. Several transcription factors [44,45] finely modulate their origin [46,47], phenotypical and functional maturation. 

Although NK cells are generally poised for rapid cytolytic activity [48], in many cases they need to be functionally differentiated [49], or primed by cytokines or other immune cells, such as DCs [50], to exert optimal effector responses. This occurs mainly for NK cells within secondary lymphoid tissues such as spleen, lymph nodes and tonsils, unlike NKs present in the peripheral blood (rev in [30]). NKs, originated both from lymphoid and non-lymphoid tissues, are able to easily reach the target organs in pathologic conditions [51]. 

NKs normally circulate in a resting phase, but they can infiltrate tissues upon cytokines activation [52,53]. Both paracrine signals (mostly cytokines) and cell-to-cell interactions are involved in the modulation of NK cell function [54]. When activated, NK cells induce apoptosis of the target cells mainly through the exocytosis of perforin and granzyme [55]. Additionally, NK cells are conceived as cytotoxic non-T lymphocytes releasing several immunoregulatory cytokines, in addition to IFN-*γ* [56,57]; these include IL-5, IL-10, IL-13, IL-22, TNF-α, GM-CSF (Granulocyte-Macrophage Colony Stimulatory Factor), macrophage inflammatory protein (MIP)-1α and 1β [58]. IFN-*γ* contributes to enhance the innate immune response and trigger the adaptive response by T lymphocytes during the priming phase, increasing both the number and activity of APCs [59,60].

**Figure 1:**
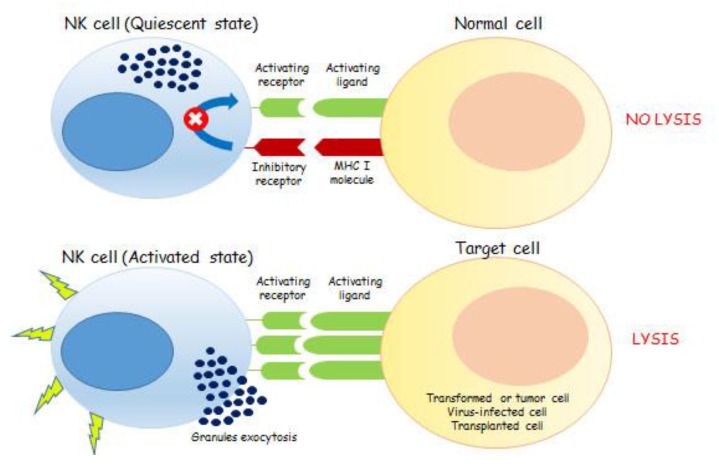
The mechanism of action of NK cells depends on the fine balance between activating and inhibitory signals. The binding of ligands, present on the cell surface and encoded by pathogens or by the host, with activating NKRs leads to NK cells activation. More specifically, NK cells express inhibitory receptors specific for MHC (Major Histocompatibility Complex class I (MHC I)) molecules on target cells. Because of direct ligand interaction, these inhibitory receptors prevent NK cell activation and killing. NK cells also express activation receptors that recognize target cell ligands and can trigger perforin-dependent natural killing. In normal cells, the integration of both activating and inhibitory signals, due to the presence of MHC I molecules, contributes to the overall state of NK quiescence. Conversely, tumor, virus-infected and transplanted cells are characterized by an enhanced expression of activatory ligands, beside the lacking or downregulation of MHC I molecules on their surface. These cells are able to drive NKs toward their activation state thus promoting target cells lysis, through the exocytosis of granzymes- and perforin-containing granules.

Unlike B and T cells, NKs do not undergo gene rearrangements thus they do not express a unique antigen recognition receptor [61,62]. The diversity in NKs depends on the specific combination of a different panel of activating and inhibitory (from two to four) NK cell receptors (NKRs) expressed on their surface (vide infra), thus originating an NK ligand-specific receptor repertoire within the total NK population (rev in [30]). In absence of antigen-selective priming, the integration and the balance of signals received from activating and inhibitory surface NKRs drives NKs toward an activated or quiescent state [63]. This balance is responsible for the exocytosis of granzymes- and perforin-containing granules, without the requirement for transcription or proliferation, leading to the apoptosis of tumors and viral-infected cells [64] (Figure 1).

NK cells are potentially able to interact with all nucleated cells through NKRs [65]. This phenomenon allows them to respond to different stimuli, to expand and differentiate generating a large repertoire of “memory-like cells” in a ligand-specific manner, i.e., 3000–35,000 functionally different NK cell subpopulations [66]. The binding of ligands, which reside on cell surface and are encoded by pathogens or by the host, with activating NKRs leads to NK cells activation. In tumor or infected cells, an enhanced expression of some of these ligands has been observed [67,68]. Additionally, NK cell activation depends also on the effect of cytokines released from APCs involved in the early host responses against pathogens (rev in [30]). 

The maturation process of NK cells is defined “licensing”, “education” or “arming”, and relies on the establishment of tolerance through the binding of inhibitory NKRs with self-MHC class I ligands, during interactions with potential target cells. As a consequence of this interaction, NKs acquire their functional competence and are driven toward more efficient effector cells [69,70]. 

## 3. Role of NK Cells in T1D

Several studies suggest that NKs could be involved in one or multiple steps of the immune-mediated attack that leads to T1D (rev in [40,71]). For their potential to interact with APCs, NKs might interfere in the priming of autoimmune responses. They might influence the downstream response, affecting the proliferation and generation of autoreactive B and T lymphocytes because of cytokine secretion (rev in [72,73]). NK cells represent the major source of IFN-*γ* (vide supra) that may significantly contribute to the excessive, uncontrolled, and unresolved autoimmune response mediated by autoreactive T cells. 

### 3.1. Animal Studies

Spontaneous animal models of T1D have always been instrumental in understanding its pathogenetic mechanisms. Among the most investigated, the NOD mouse most closely resembles human disease (rev in [74]). In addition, Bio Breeding diabetes-prone [BB], Komeda [KDP], LEW 1AR1/-iddm rat strains were investigated (rev in [74]). Nevertheless, evidences for the role of NKs addressing either a promoting or a protective effect in disease onset and development were only reported in NOD mice (Table 1) [75,76,77,78]. We need however to point out that NOD mice are characterized by an unusual composition in the genomic regions that influence NK cell activity; thus, whether this murine model can indeed foresee the NK cell behavior in human T1D is still a debated issue [11]. 

Among the initial evidences, beside their recovery in the pancreas of non-obese diabetic (NOD) [79] and BDC/NOD mice [76], Flodstrom’s group (2002) observed the destructive effect of NKs within NOD pancreatic cells [75]*.* Further, in the study by Poirot et al. (2004) [76] preferential NK cell recruitment was found in the aggressive insulitic lesions of BDC2.5/B6.H-2^g7^ versus BDC2.5/NOD animals [76]. NK cell recruitment was enhanced in the aggressive insulitis of BDC2.5/NOD mice following CTLA-4 (cytotoxic T lymphocyte antigen 4) blockade, indicating the early involvement of this subset in β cell destruction [76]. NKs with a specific phenotype (higher levels of CD69 and CD25, lower levels of L-selectin (CD62L)) were identified in the pancreas of NOD mice in the study performed by Brauner and colleagues (2010) [78]. A decreased activity emerged in pancreatic compared to spleen NKs, thus confirming their putative involvement in the inflammatory process [Brauner 2010]. Pancreatic NKs initially could mediate proinflammatory effector functions, potentially contributing to organ-specific autoimmunity, but later on they could become hyporesponsive because of exhaustion or regulation [78]. Another study evidencing a protective role of NK*s* in the development of T1D was performed by Lee and colleagues (2004), showing downregulation of autoreactive cytotoxic T lymphocytes (CTL) that limits β cell destruction [77]. This may address NK cells as possible targets of a novel approach for the prevention of autoimmune diabetes [77].

### 3.2. Human Studies

Ambiguous results on NK cell aberrations have been reported in T1D patients (Table 2) with the limitation regarding the use of peripheral blood samples that might not precisely reflect the ongoing process in the infiltrated pancreatic islets [80]. Some investigations described a reduced NK cell number, altered lytic activity and changes in the expression of activating receptors in T1D peripheral blood mononuclear cells (PBMC) (rev in [30,81]), whereas other studies highlighted the association between stage of disease and the presence of transitory cellular differences [82,83]. Functional abnormalities have also been reported in NK cells of T1D patients. A reduced lytic capacity, as determined by cytotoxicity assays, was also reported, but not universally confirmed [84,85], both in recently diagnosed and/or long-standing T1D patients [82,83]. 

Defects in NK cell frequency and activation were found in patients with latent autoimmune diabetes in adults (LADA) [86]. Accordingly to previous results from recent-onset T1D patients [40,85,87], a consistent reduction in NK cell frequency was shown in the peripheral blood of LADA patients, probably correlated with a parallel NK cell increase in the draining lymph nodes and pancreas [86]. Although T1D and LADA showed a different activating NK cell phenotype, the finding of reduced NK cell frequency allows to hypothesize comparable immunological alterations, i.e., an attenuated NK cytotoxic phenotype in the periphery and altered NKR expression (rev in [30]). As opposite to studies underlying that the disease onset is marked by a slight reduction in NK cells, Rodacki et al. (2007) found that this subset is unusually activated in some patients with IFN-*γ* expression [40]. Lower expression of NK p30/p46 activating receptor molecules was attributed to prolonged hyperglycemia. Decreased expression of NKG2D was detected in diabetic patients independently of disease duration as well as an increased frequency of KIR gene haplotypes [40]. 

An intriguing hypothesis concerning T1D development is that T1D susceptible subjects could manifest a higher predisposition to viral infections due to an aberrant responsiveness of pancreatic cells to IFN-*γ* (rev in [81]). The autoimmune process might be triggered by the release of autoantigens due to NK cell activity. Autoantigens could stimulate autoreactive T cells and initiate disease. In recent onset T1D patients, Dotta and colleagues (2007) [80] observed several cases of infections of β cells due to Coxsackie B4 virus, leading to the infiltration of NKs into pancreatic islets, with consequent insulitis and β cell destruction. 

## 4. Molecular Mechanisms of NKs in T1D

Although NK cells are believed to play a crucial role in diabetes development [76,88], the mechanisms underlying their function are not fully clarified. The activity and function of NK cells are dependent on the resulting effect of either activating and inhibitory receptors interactions (vide supra*)* (rev in [30,33,89]). Typically, NK cell receptors are germline encoded, as opposite to T and B antigen-specific receptors, which are somatically recombined. In particular NK cell activating receptors are of limited repertoire (rev in [30,33,90]) (vide supra); among these, the natural cytotoxicity receptor (NCR) family, including human NKp30 [91], NKp44 [92], and NKp46 [93] Ig-like proteins is mostly represented. NKp46 and NKp30 are constitutively expressed by all NK cells subpopulations, while NKp44 exclusively upon activation (rev in [9,30,33]). NKp46, whose codifying gene is located in the leukocyte-receptor complex on human chromosome 19, is considered the most specific NK cell marker and is the only NCR with a murine orthologue, namely NCR1 [89]. In addition to NCRs, NK cells express several other activating receptors, including NKG2D, which recognizes stress-induced ligands expressed by cancerous, virally infected, and other stressed cells [89]. While the NCRs are expressed almost exclusively on NK cells, NKG2D is also expressed on lymphocytes, such as CD8+ T cells [94].

NK cell subpopulations may elicit a different response upon encountering a potential target cell either killing or inducing unresponsiveness (Figure 1). Nevertheless, the underlying mechanism of NK cell activation relies on triggering activating signals, originated by their corresponding receptors, integrated with repressive signals from inhibitory receptors (vide supra). 

Although the involvement of NKp46 in the defense against pathogens is well-recognized [95], Gur and colleagues (2010) demonstrated for the first time this phenomenon in T1D pathogenesis [94]. They found that NKp46 and the orthologue NCR1 recognize human and murine pancreatic β cells; this specific interaction is able to activate NK cell degranulation upon contact with murine β cells [94]. In a subsequent study, Yossef and colleagues (2015) [96] developed an antibody-mediated strategy to unravel NKp46 function in T1D. Mice treated with NCR1.15 mAb, that specifically binds mNKp46, showed an impairment in NK function both in vivo and in vitro without NK depletion. Furthermore, repeated treatments with NCR1.15 inhibited development of diabetes in low-dose streptozotocin diabetes (LDSTZ) model and in NOD mice, suggesting a novel putative therapeutic strategy for early insulitis [96]. In an interesting study by Wang and colleagues (2015) [97], higher numbers of circulating NKp46^+^ NK cells emerged in T1D patients compared to healthy controls, suggesting a key role of NKp46^+^ activated NKs in the pathogenesis of LADA. Furthermore, LADA patients were characterized by a significantly enhanced frequency of INF-*γ* secreting NK cells, confirming a direct involvement of the NK subpopulation in DM onset. 

C-type lectin NKG2D is a relevant activating receptor for recognition of stressed, infected, or transformed cells [98]. In the study performed by Ogasawara and colleagues (2003) [99] in NOD mice, NK cells showed a reduced NKG2D-dependent activity, suggesting this could contribute to disease pathogenesis. 

In two distinct studies [40,99], NK cell frequencies were found reduced in T1D patients. This feature was correlated with an impaired responsiveness to IL-2/IL-15 stimulation [100], suggesting that cell-intrinsic mechanisms may be responsible for their own reduced frequencies. Murine NK cell homeostasis and NKG2D function are probably co-regulated by the coupling of NKG2D and IL-15 receptors; thus, in T1D, both defective cytokine responsiveness and NKG2D functionality of NK cells might rely on a common pathway. As regards, the fact that NKG2D deficient mice possess aberrant NK cell number, proliferation and apoptosis supports the NKG2D’s crucial role in NK cell homeostasis [101]. The involvement of NKG2D in the onset of T1D was also addressed by Van Belle and collaborators (2009) [102] with the finding of altered expression of NKG2D ligands on β cells. In detail, downregulation of NKG2D on circulating NK cells and CD8^+^ T cells in the spleen and pancreatic lymph nodes is able to reverse recent-onset diabetes in NOD mice [103]. 

The expression of the activatory NKG2D receptor was found significantly enhanced in T1D patients [86], as opposite to the results obtained by Ogasawara and colleagues (2003) in NOD mice [99] and by Rodacki and colleagues (2007) [40] (vide supra). The overexpression of NKG2D could be involved in a lower progression of LADA into insulin-dependent diabetes. 

In a recent study performed by Shalaby and collaborators (2017) [104], a significant downregulation of the KLRC3 (kinase light chain 3) gene (codifying for NKG2E receptor) emerged in T1D patients compared to healthy controls. Downregulation of the KLRC3 gene may result in abnormal NKG2E receptor expression and, consequently, abnormal NK cells function. Normally NKG2E forms heterodimeric complexes with CD94 and binds HLA-E, a MHC Ib protein involved in T1D pathogenesis [40].

Recently, the existence of “memory-like” NK cell subsets has been demonstrated emerging upon viral infections such as HCMV or in contact hypersensitivity conditions evoked by haptens. Several reports suggest that NK cell subsets may behave differently in response to distinct pathogens (rev in [30]). As regards T1D, we recently depicted a functionally and phenotypically skewed CD3^−^CD8^dull^CD56^+^ “memory like” NK subset in the mainstream of newly diagnosed T1D patients [33]. This was expandable upon GAD65 AA 114–122 epitope stimulation and identified by HLA-class I pentamers binding through the Ig-like transcript 2 (ILT2) receptor at inhibitory activity. The pathogenetic significance of this “memory-like NK cell subset” in diabetics remains to be elucidated. 

## 5. Conclusions and Future Perspectives

Although many details of the complex pathophysiology of T1D have been completely clarified, several aspects remain unknown. Observing the complex puzzle of the different immunocytes involved in T1D pathogenesis, in addition to the traditional view of the concerted action of T, B lymphocytes, macrophages and DCs, NK cells appear as a still “undiscovered” player to be investigated. Although initially identified as innate lymphocytes (rev in [30]), they show characteristics of the adaptive immune system (vide supra). Thus, this subset is reasonably implicated in autoimmunity and tissue inflammation [105,106]. Abnormalities in the number and activity of NK cells, leading to instability of immune system and uncontrolled proliferation of certain immunotypes [81], have been reported both in animal models and T1D patients (vide supra). Some of these alterations are linked to its onset while others seem to be a consequence of the disease [71], with the development of severe clinical implications. 

**Figure 2:**
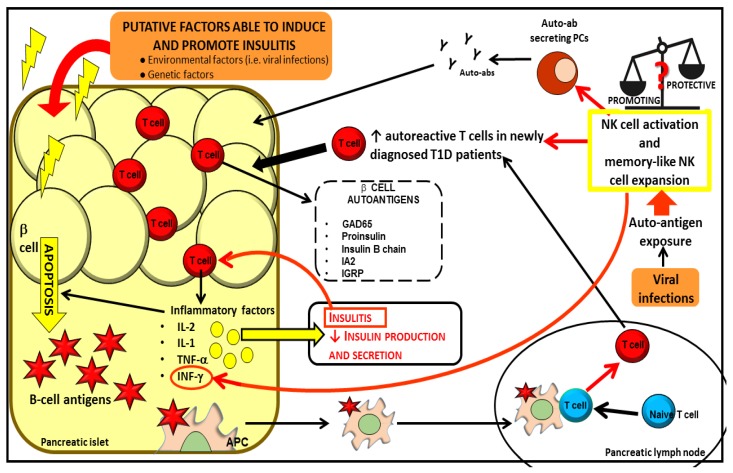
Both genetic and environmental factors cause β cell apoptosis leading to β cell antigen release. Self-antigens induce APC activation. Activated APCs promote the activation of naïve T cells in the pancreatic lymph nodes. Autoreactive T cells infiltrate pancreatic islet and release pro-inflammatory factors promoting insulitis, amplifying the apoptotic process and reducing insulin production and secretion. NKs could be involved in one or multiple steps of the immune-mediated attack that lead to T1D, although their protective or promoting role in this process remains to be elucidated. NKs can affect the proliferation and generation of T and B autoreactive lymphocytes. Mature B cells differentiate in plasma cells (PCs) producing autoantibodies, which, in turn, further contribute to the tissue inflammatory process and destruction. NKs cells can also act as “memory like cells” expanding upon auto-antigenic exposure in response to viruses.

Nevertheless, from the literature evidences above discussed, the exact role of NKs in T1D pathogenesis is still ambiguous [107], probably due to the extent of different NK subsets presenting functional differences; protection or exacerbation of the autoimmune disease may be due to disequilibrium between the different subsets [108]. Thus, a deeper investigation of functional NK subsets and their inflammatory and regulatory phenotypes could unravel their functional effect in T1D [107]. This concept is strengthened by the recent observation that among their pleiotropic features, contrary to dogma, NKs cells can also act as “memory like cells” expanding upon autoantigenic exposure [33] as already demonstrated in response to viruses [109]. This opens new research pathways that may lead to elucidate their characterization and the appropriate balance of activating and inhibitory receptors in different stages of disease in this particular subset. This also may lead to discover prevalent functional effects in different stages of disease leading to unexpected subtle immunomodulatory effects either in the peripheral blood or at insulitis level. Furthermore, memory like NK cells could also be expanded under autoantigen-nonspecific bystander conditions as viral infections that may be contributing to the disease pathogenesis [33] (Figure 2).

Certainly, future studies are necessary to clarify mechanisms of interaction with APC, T cells and target pancreatic β-cells that are ultimately destroyed [84]. Understanding the exact role of NK cells in the pathogenesis and progression of T1D and which particular NK subsets are involved could help determine if they can be manipulated therapeutically in autoimmune diseases [108] to support the development of novel immune-based strategies. These would have the aim to reduce T1D risk or delay disease onset. Furthermore, the elucidation of NK abnormalities and their putative involvement in the risk of infections or neoplasia in T1D patients could be useful to prevent the occurrence of these conditions during disease progression.

## Figures and Tables

**Table 1:** Non-obese diabetic (NOD) animal studies supporting the promoting or protective role of NKs towards T1D onset.

Promoting Effects	Protective Effects
Pancreatic β cell destruction [75] NK cells are increased in the aggressive insulitic lesions of BDC2.5/B6-H-2^g7^ than BDC2.5/NOD mice [76] NK cell early participation of aggressive pancreatic lesions in BDC2.5/NOD mice treated by CTLA-4 blockade [76] NK infiltration in the pancreas contributing to inflammatory processes [78]	Down-regulation of autoreactive CTL NK-mediated limits pancreatic β cell destruction [77]

**Table 2:** NK cell studies in T1D patients.

Coxsachie B4 β cell infection and NK insulitis in newly diagnosed T1D patients [80] Dichotomy between islet killer cell and NK cell activities [82] Defective NK cell cytotoxicity [83] Increased IFN and IL-2-induced NK activities in T1D lymphocytes than in controls [84] Genetically determined reduced number of NK cells in T1D with increased activity at onset [85] Impaired NK functionality towards target cells and enhanced islet killing activity [86] Reduced number of NKs in T1D patients [87] NK cell activation with IFN γ expression, lower expression of NK p30/46 and NKG2D, increased frequency of KIR haplotypes [40]

## Abbreviations

DM	Diabetes Mellitus
T1D	Type 1 Diabetes
T2D	Type 2 Diabetes
GDM	Gestational Diabetes Mellitus
HLA	Human leukocyte antigen
*PTPN22*	Protein tyrosine phosphatase non-receptor type 22
*IL-2RA*	Interleukin 2-receptor alpha
GWAS	Genome wide association studies
SNP	Single nucleotide polymorphism
GAD65	Glutamic acid decarboxylase isoform 65
IA-2	Tyrosine phosphatase-like insulinoma-associated antigen
IGRP	Islet-specific glucose-6—phosphatase catalytic subunit-related protein
TCR	T cell receptor
Th	T helper
Treg	T regulatory cells
IL	Interleukin
TNF-α	Tumor necrosis factor α
IFN	Interferon
Foxp3	Forkhead box P3
BCR	B cell receptor
NO	Nitric oxide
DC	Dendritic cells
APC	Antigen-presenting cell
pDC	Plasmocytoid dendritic cell
NK	Natural killer
NKT	NK-like T
KIR	Killer cell immunoglobulin-like receptor
HSC	Hematopoietic stem cell
GM-CSF	Granulocyte-macrophage colony stimulating factor
MIP	Macrophage inflammatory protein
NKR	NK receptor
MHC	Major Histocompatibility Complex
MHC I	Major Histocompatibility Complex class I
NOD	Non obese diabetic
CTLA-4	Cytotoxic T lymphocyte antigen 4
CD62L	L-selecting
CTL	Cytotoxic T lymphocyte
PBMC	Peripheral blood mononuclear cells
LADA	Latent Autoimmune Diabetes in Adults
NCR	Natural cytotoxicity receptor
KLRC3	Kinase light chain 3
ILT2	Ig-like transcript 2
HCMV	Human cytomegalovirus
PC	Plasma cell

## References

[1] Kerner W., Bruckel J., German Diabetes Association (2014). Definition, classification and diagnosis of diabetes mellitus. Exp. Clin. Endocrinol. Diabetes.

[2] American Diabetes Association (2009). Diagnosis and Classification of Diabetes Mellitus. Diabetes Care.

[3] Bluestone J.A., Herold K., Eisenbarth G. (2010). Genetics, pathogenesis and clinical interventions in type 1 diabetes. Nature.

[4] Liese A.D., D’Agostino R.B., Hamman R.F., Kilgo P.D., Lawrence J.M., Liu L.L., Loots B., Linder B., Marcovina S., Rodriguez B. (2006). The burden of diabetes mellitus among US youth: Prevalence estimates from the SEARCH for Diabetes in Youth Study. Pediatrics.

[5] Dabelea D., Bell R.A., D’Agostino R.B., Imperatore G., Johansen J.M., Linder B., Liu L.L., Loots B., Marcovina S., Mayer-Davis E.J. (2007). Incidence of diabetes in youth in the United States. JAMA.

[6] Janeway C.A., Travers P., Walport M., Shlomchik M.J. (2005). Immunobiology.

[7] Fierabracci A. (2016). Type 1 Diabetes in Autoimmune Polyendocrinopathy-Candidiasis-Ectodermal Dystrophy Syndrome (APECED): A “Rare” Manifestation in a “Rare” Disease. Int. J. Mol. Sci..

[8] Marrack P., Kappler J., Kotzin B.L. (2001). Autoimmune disease: Why and where it occurs. Nat. Med..

[9] Pociot F. (2017). Type 1 diabetes genome-wide association studies: Not to be lost in translation. Clin. Transl. Immunol..

[10] Cabrera S.M., Chen Y.G., Hagopian W.A., Hessner M.J. (2016). Blood-based signatures in type 1 diabetes. Diabetologia.

[11] Parisi L., Bassani B., Tremolati M., Gini E., Farronato G., Bruno A. (2017). Natural Killer Cells in the Orchestration of Chronic Inflammatory Diseases. J. Immunol. Res..

[12] Ziegler A.G., Rewers M., Simell O., Simell T., Lempainen J., Steck A., Winkler C., Ilonen J., Veijola R., Knip M. (2013). Seroconversion to multiple islet autoantibodies and risk of progression to diabetes in children. JAMA.

[13] Gepts W. (1965). Pathologic anatomy of the pancreas in juvenile diabetes mellitus. Diabetes.

[14] Pugliese A. (2016). Insulitis in the pathogenesis of Type 1 diabetes. Pediatr. Diabetes.

[15] Giuliani L., Mele R., Di Giovine M., Altieri L., Crinò A., Ravà L., Fierabracci A. (2009). Detection of GAD65 Autoreactive T-Cells by HLA Class I Tetramers in Type 1 Diabetic Patients. J. Biomed. Biotechnol..

[16] Nagafuchi S. (2010). The role of B cells in regulating the magnitude of immune response. Microbiol. Immunol..

[17] Arif S., Leete P., Nguyen V., Marks K., Nor N.M., Estorninho M., Kronenberg-Versteeg D., Bingley P.J., Todd J.A., Guy C. (2014). Blood and islet phenotypes indicate immunological heterogeneity in type 1 diabetes. Diabetes.

[18] Fierabracci A., Ayroldi E. (2011). Experimental strategies in autoimmunity: Antagonists of cytokines and their receptors, nanocarriers, inhibitors of immunoproteasome, leukocyte migration and protein kinases. Curr. Pharm. Des..

[19] Kuriya G., Uchida T., Akazawa S., Kobayashi M., Nakamura K., Satoh T., Horie I., Kawasaki E., Yamasaki H., Yu L. (2013). Double deficiency in IL-17 and IFN-γ signalling significantly suppresses the development of diabetes in the NOD mouse. Diabetologia.

[20] Ferraro A., Socci C., Stabilini A., Valle A., Monti P., Piemonti L., Nano R., Olek S., Maffi P., Scavini M. (2011). Expansion of Th17 cells and functional defects in T regulatory cells are key features of the pancreatic lymph nodes in patients with type 1 diabetes. Diabetes.

[21] Perri V., Russo B., Crinò A., Schiaffini R., Giorda E., Cappa M., Rosado M.M., Fierabracci A. (2015). Expression of PD-1 Molecule on Regulatory T Lymphocytes in Patients with Insulin-Dependent Diabetes Mellitus. Int. J. Mol. Sci..

[22] Clark M., Kroger C.J., Tisch R.M. (2017). Type 1 Diabetes: A Chronic Anti-Self-Inflammatory Response. Front. Immunol..

[23] Lee J.H., Noh J., Noh G., Choi W.S., Cho S., Lee S.S. (2011). Allergen-specific transforming growth factor-beta-producing CD19+CD5+ regulatory B-cell (Br3) responses in human late eczematous allergic reactions to cow’s milk. J. Interferon Cytokine Res..

[24] Yokosuka T., Saito T. (2010). The immunological synapse, TCR microclusters, and T cell activation. Curr. Top. Microbiol. Immunol..

[25] Yang J., Reth M. (2010). The dissociation activation model of B cell antigen receptor triggering. FEBS Lett..

[26] Lee K.U., Amano K., Yoon J.W. (1988). Evidence for initial involvement of macrophages in development of insulitis in NOD mice. Diabetes.

[27] Carrero J.A., McCarthy D.P., Ferris S.T., Wan X., Hu H., Zinselmeyer B.H., Vomund A.N., Unanue E.R. (2017). Resident macrophages of pancreatic islets have a seminal role in the initiation of autoimmune diabetes of NOD mice. Proc. Natl. Acad. Sci. USA.

[28] Mbongue J.C., Nieves H.A., Torrez T.W., Langridge W.H.R. (2017). The Role of Dendritic Cell Maturation in the Induction of Insulin-Dependent Diabetes Mellitus. Front. Immunol..

[29] Allen J.S., Pang K., Skowera A., Ellis R., Rackham C., Lozanoska-Ochser B., Tree T., Leslie R.D.G., Tremble J.M., Dayan CM. (2009). Plasmacytoid dendritic cells are proportionally expanded at diagnosis of type 1 diabetes and enhance islet autoantigen presentation to T-cells through immune complex capture. Diabetes.

[30] Gianchecchi E., Delfino D.V., Fierabracci A. (2017). NK cells in autoimmune diseases: Linking innate and adaptive responses. Autoimmun. Rev..

[31] Perricone R., Perricone C., De Carolis C., Shoenfeld Y. (2008). NK cells in autoimmunity: A two-edg’d weapon of the immune system. Autoimmun. Rev..

[32] Horwitz D.A., Gray J.D., Ohtsuka K., Hirokawa M., Takahashi T. (1997). The immunoregulatory effects of NK cells: The role of TGF-beta and implications for autoimmunity. Immunol. Today.

[33] Perri V., Gianchecchi E., Cifaldi L., Pellegrino M., Giorda E., Andreani M., Cappa M., Fierabracci A. (2017). Identification of GAD65 AA 114-122 reactive ‘memory-like’ NK cells in newly diagnosed Type 1 diabetic patients by HLA-class I pentamers. PLoS ONE.

[34] Nakata S., Imagawa A., Miyata Y., Yoshikawa A., Kozawa J., Okita K., Funahashi T., Nakamura S., Matsubara K., Iwahashi H. (2013). Low gene expression levels of activating receptors of natural killer cells (NKG2E and CD94) in patients with fulminant type 1 diabetes. Immunol. Lett..

[35] Kiessling R., Klein E., Pross H., Wigzell H. (1975). “Natural” killer cells in the mouse. II. Cytotoxic cells with specificity for mouse Moloney leukemia cells. Characteristics of the killer cell. Eur. J. Immunol..

[36] Kiessling R., Klein E., Wigzell H. (1975). “Natural” killer cells in the mouse. I. Cytotoxic cells with specificity for mouse Moloney leukemia cells. Specificity and distribution according to genotype. Eur. J. Immunol..

[37] Herberman R.B., Nunn M.E., Holden H.T., Lavrin D.H. (1975). Natural cytotoxic reactivity of mouse lymphoid cells against syngeneic and allogeneic tumors. II. Characterization of effector cells. Int. J. Cancer.

[38] Trinchieri G., Perussia B., Santoli D., Cerottini J.C. (1979). Human natural killer cells. Transplant. Proc..

[39] Atkinson M.A. (2012). The pathogenesis and natural history of type 1 diabetes. Cold Spring Harb. Perspect. Med..

[40] Rodacki M., Svoren B., Butty V., Besse W., Laffel L., Benoist C., Mathis D. (2007). Altered natural killer cells in type 1 diabetic patients. Diabetes.

[41] Cooper M.A., Elliott J.M., Keyel P.A., Yang L., Carrero J.A., Yokoyama W.M. (2009). Cytokine-induced memory-like natural killer cells. Proc. Natl. Acad. Sci. USA.

[42] Yu J., Mao H.C., Wei M., Hughes T., Zhang J., Park I.K., Liu S., McClory S., Marcucci G., Trotta R. (2010). CD94 surface density identifies a functional intermediary between the CD56bright and CD56dim human NK-cell subsets. Blood.

[43] Mandal A., Viswanathan C. (2015). Natural killer cells: In health and disease. Hematol. Oncol. Stem Cell Ther..

[44] Ferlazzo G., Pack M., Thomas D., Paludan C., Schmid D., Strowig T., Bougras G., Muller W.A., Moretta L., Münz C. (2004). Distinct roles of IL-12 and IL-15 in human natural killer cell activation by dendritic cells from secondary lymphoid organs. Proc. Natl. Acad. Sci. USA.

[45] Freud A.G., Becknell B., Roychowdhury S., Mao H.C., Ferketich A.K., Nuovo G.J., Hughes T.L., Marburger T.B., Sung J., Baiocchi R.A. (2005). A human CD34(+) subset resides in lymph nodes and differentiates into CD56bright natural killer cells. Immunity.

[46] Barton K., Muthusamy N., Fischer C., Ting C.N., Walunas T.L., Lanier L.L., Leiden J.M. (1998). The Ets-1 transcription factor is required for the development of natural killer cells in mice. Immunity.

[47] Ma A., Koka R., Burkett P. (2006). Diverse functions of IL-2, IL-15, and IL-7 in lymphoid homeostasis. Annu. Rev. Immunol..

[48] Bezman N.A., Kim C.C., Sun J.C., Min-Oo G., Hendricks D.W., Kamimura Y., Best J.A., Goldrath A.W., Lanier L.L. (2012). Molecular definition of the identity and activation of natural killer cells. Nat. Immunol..

[49] Vivier E. (2006). What is natural in natural killer cells?. Immunol. Lett..

[50] Lucas M., Schachterle W., Oberle K., Aichele P., Diefenbach A. (2007). Dendritic cells prime natural killer cells by trans-presenting interleukin 15. Immunity.

[51] Cudkowicz G., Stimpfling J.H. (1964). Hybrid resistance to parental marrow grafts: Association with the K region of H-2. Science.

[52] Fogler W.E., Volker K., McCormick K.L., Watanabe M., Ortaldo J.R., Wiltrout R.H. (1996). NK cell infiltration into lung, liver, and subcutaneous B16 melanoma is mediated by VCAM-1/VLA-4 interaction. J. Immunol..

[53] Glas R., Franksson L., Une C., Eloranta M.L., Ohlén C., Orn A., Kärre K. (2000). Recruitment and activation of natural killer (NK) cells in vivo determined by the target cell phenotype. An adaptive component of NK cell mediated responses. J. Exp. Med..

[54] Spada R., Rojas J.M., Barber D.F. (2015). Recent findings on the role of natural killer cells in the pathogenesis of systemic lupus erythematosus. J. Leukoc. Biol..

[55] Smith H.R., Heusel J.W., Mehta I.K., Kim S., Dorner B.G., Naidenko O.V., Iizuka K., Furukawa H., Beckman D.L., Pingel J.T. (2002). Recognition of a virus-encoded ligand by a natural killer cell activation receptor. Proc. Natl. Acad. Sci. USA.

[56] Vitale M., Della Chiesa M., Carlomagno S., Romagnani C., Thiel A., Moretta L., Moretta A. (2004). The small subset of CD56brightCD16-natural killer cells is selectively responsible for both cell proliferation and interferon-gamma production upon interaction with dendritic cells. Eur. J. Immunol..

[57] Moretta L., Locatelli F. (2016). Innate lymphoid cells in normal and disease: An introductory overview. Immunol. Lett..

[58] Backstrom E., Kristensson K., Ljunggren H.G. (2004). Activation of natural killer cells: Underlying molecular mechanisms revealed. Scand. J. Immunol..

[59] Strowig T., Brilot F., Munz C. (2008). Noncytotoxic functions of NK cells: Direct pathogen restriction and assistance to adaptive immunity. J. Immunol..

[60] Yokoyama W.M. (2008). Mistaken notions about natural killer cells. Nat. Immunol..

[61] Shinkai Y., Rathbun G., Lam K.P., Oltz E.M., Stewart V., Mendelsohn M., Charron J., Datta M., Young F., Stall A.M. (1992). RAG-2-deficient mice lack mature lymphocytes owing to inability to initiate V(D)J rearrangement. Cell.

[62] Mombaerts P., Iacomini J., Johnson R.S., Herrup K., Tonegawa S., Papaioannou V.E. (1992). RAG-1-deficient mice have no mature B and T lymphocytes. Cell.

[63] Lanier L.L. (2008). Up on the tightrope: Natural killer cell activation and inhibition. Nat. Immunol..

[64] Pegram H.J., Andrews D.M., Smyth M.J., Darcy P.K., Kershaw M.H. (2011). Activating and inhibitory receptors of natural killer cells. Immunol. Cell. Biol..

[65] Vosshenrich C.A., Samson-Vill´eger S.I., Di Santo J.P. (2005). Distinguishing features of developing natural killer cells. Curr. Opin. Immunol..

[66] Horowitz A., Strauss-Albee D.M., Leipold M., Kubo J., Nemat-Gorgani N., Dogan O.C., Dekker C.L., Mackey S., Maecker H., Swan G.E. (2013). Genetic and environmental determinants of human NK cell diversity revealed by mass cytometry. Sci. Transl. Med..

[67] Smyth M.J., Cretney E., Kelly J.M., Westwood J.A., Street S.E., Yagita H., Takeda K., van Dommelen S.L., Degli-Esposti M.A., Hayakawa Y. (2005). Activation of NK cell cytotoxicity. Mol. Immunol..

[68] Guerra N., Tan Y.X., Joncker N.T., Choy A., Gallardo F., Xiong N., Knoblaugh S., Cado D., Greenberg N.M., Raulet D.H. (2008). NKG2D-deficient mice are defective in tumor surveillance in models of spontaneous malignancy. Immunity.

[69] Thomas L.M. (2015). Current perspectives on natural killer cell education and tolerance: Emerging roles for inhibitory receptors. Immunotargets Ther..

[70] Raulet D.H., Vance R.E. (2006). Self-tolerance of natural killer cells. Nat. Rev. Immunol..

[71] Rodacki M., Milech A., de Oliveira J.E. (2006). NK cells and type 1 diabetes. Clin. Dev. Immunol..

[72] French A.R., Yokoyama W.M. (2004). Natural killer cells and autoimmunity. Arthritis Res. Ther..

[73] Sinkovics J.G., Horvath J.C. (2005). Human natural killer cells: A comprehensive review. Int. J. Oncol..

[74] Al-Awar A., Kupai K., Veszelka M., Szűcs G., Attieh Z., Murlasits Z., Török S., Pósa A., Varga C. (2016). Experimental diabetes mellitus in different animal models. Diabetes Res..

[75] Flodström M., Maday A., Balakrishna D., Cleary M.M., Yoshimura A., Sarvetnick N. (2002). Target cell defense prevents the development of diabetes after viral infection. Nat. Immunol..

[76] Poirot L., Benoist C., Mathis D. (2004). Natural killer cells distinguish innocuous and destructive forms of pancreatic islet autoimmunity. Proc. Natl. Acad. Sci. USA.

[77] Lee I.F., Qin H., Trudeau J., Dutz J., Tan R. (2004). Regulation of autoimmune diabetes by complete Freund’s adjuvant is mediated by NK Cells. J. Immunol..

[78] Brauner H., Elemans M., Lemos S., Broberger C., Holmberg D., Flodström-Tullberg M., Kärre K., Höglund P. (2010). Distinct phenotype and function of NK cells in the pancreas of nonobese diabetic mice. J. Immunol.

[79] Miyazaki A., Hanafusa T., Yamada K., Miyagawa J., Fujino-Kurihara H., Nakajima H., Nonaka K., Tarui S. (1985). Predominance of T lymphocytes in pancreatic islets and spleen of pre-diabetic non-obese diabetic (NOD) mice: A longitudinal study. Clin. Exp. Immunol..

[80] Dotta F., Censini S., van Halteren A.G., Marselli L., Masini M., Dionisi S., Mosca F., Boggi U., Muda A.O., Del Prato S. (2007). Coxsackie B4 virus infection of beta cells and natural killer cell insulitis in recent-onset type 1 diabetic patients. Proc. Natl. Acad. Sci. USA.

[81] Popko K., Górska E. (2015). The role of natural killer cells in pathogenesis of autoimmune diseases. Cent. Eur. J. Immunol..

[82] Negishi K., Waldeck N., Chandy G., Buckingham B., Kershnar A., Fisher L., Gupta S., Charles M.A. (1986). Natural killer cell and islet killer cell activities in type 1 (insulin-dependent) diabetes. Diabetologia.

[83] Lorini R., Moretta A., Valtorta A., D’Annunzio G., Cortona L., Vitali L., Bozzola M., Severi F. (1994). Cytotoxic activity in children with insulin-dependent diabetes mellitus. Diabetes Res. Clin. Pract..

[84] Nair M.P., Lewis E.W., Schwartz S.A. (1986). Immunoregulatory dysfunctions in type I diabetes: Natural and antibody-dependent cellular cytotoxic activities. J. Clin. Immunol..

[85] Akesson C., Uvebrant K., Oderup C., Lynch K., Harris R.A., Lernmark A., Agardh C.D., Cilio C.M. (2010). Altered natural killer (NK) cell frequency and phenotype in latent autoimmune diabetes in adults (LADA) prior to insulin deficiency. Clin. Exp. Immunol..

[86] Hussain M.J., Alviggi L., Millward B.A., Leslie R.D., Pyke D.A., Vergani D. (1987). Evidence that the reduced number of natural killer cells in type 1 (insulin-dependent) diabetes may be genetically determined. Diabetologia.

[87] Wilson R.G., Anderson J., Shenton B.K., White M.D., Taylor R.M., Proud G. (1986). Natural killer cells in insulin dependent diabetes mellitus. BMJ.

[88] Alba A., Planas R., Clemente X., Carrillo J., Ampudia R., Puertas M.C., Pastor X., Tolosa E., Pujol-Borrell R., Verdaguer J. (2008). Natural killer cells are required for accelerated type 1 diabetes driven by interferon-β. Clin. Exp. Immunol..

[89] Enk J., Mandelboim O. (2014). The role of natural cytotoxicity receptors in various pathologies: Emphasis on type I diabetes. Front. Immunol..

[90] Paust S., von Andrian U.H. (2011). Natural killer cell memory. Nat. Immunol..

[91] Pende D., Parolini S., Pessino A., Sivori S., Augugliaro R., Morelli L., Marcenaro E., Accame L., Malaspina A., Biassoni R. (1999). Identification and molecular characterization of NKp30, a novel triggering receptor involved in natural cytotoxicity mediated by human natural killer cells. J. Exp. Med..

[92] Vitale M., Bottino C., Sivori S., Sanseverino L., Castriconi R., Marcenaro E., Augugliaro R., Moretta L., Moretta A. (1998). NKp44, a novel triggering surface molecule specifically expressed by activated natural killer cells, is involved in non-major histocompatibility complex-restricted tumor cell lysis. J. Exp. Med..

[93] Sivori S., Vitale M., Morelli L., Sanseverino L., Augugliaro R., Bottino C., Moretta L., Moretta A. (1997). p46, a novel natural killer cell-specific surface molecule that mediates cell activation. J. Exp. Med..

[94] Gur C., Porgador A., Elboim M., Gazit R., Mizrahi S., Stern-Ginossar N., Achdout H., Ghadially H., Dor Y., Nir T. (2010). The activating receptor NKp46 is essential for the development of type 1 diabetes. Nat. Immunol..

[95] Satoh-Takayama N., Dumoutier L., Lesjean-Pottier S., Ribeiro V.S., Mandelboim O., Renauld J.C., Vosshenrich C.A., Di Santo J.P. (2009). The natural cytotoxicity receptor NKp46 is dispensable for IL-22-mediated innate intestinal immune defense against Citrobacter rodentium. J. Immunol..

[96] Yossef R., Gur C., Shemesh A., Guttman O., Hadad U., Nedvetzki S., Miletić A., Nalbandyan K., Cerwenka A., Jonjic S. (2015). Targeting natural killer cell reactivity by employing antibody to NKp46: Implications for type 1 diabetes. PLoS ONE.

[97] Wang Y., Yuan W., Guo H., Jiang Y. (2015). High frequency of activated NKp46(+) natural killer cells in patients with new diagnosed of latent autoimmune diabetes in adults. Autoimmunity.

[98] Raulet DH. (2003). Roles of the NKG2D immunoreceptor and its ligands. Nat. Rev. Immunol..

[99] Ogasawara K., Hamerman J.A., Hsin H., Chikuma S., Bour-Jordan H., Chen T., Pertel T., Carnaud C., Bluestone J.A., Lanier L.L. (2003). Impairment of NK cell function by NKG2D modulation in NOD mice. Immunity.

[100] Qin H., Lee I.F., Panagiotopoulos C., Wang X., Chu A.D., Utz P.J., Priatel J.J., Tan R. (2011). Natural killer cells from children with type 1 diabetes have defects in NKG2D-dependent function and signaling. Diabetes.

[101] Horng T., Bezbradica J.S., Medzhitov R. (2007). NKG2D signaling is coupled to the interleukin 15 receptor signaling pathway. Nat. Immunol..

[102] Van Belle T.L., von Herrath M.G. (2009). The role of the activating receptor NKG2D in autoimmunity. Mol. Immunol..

[103] Van Belle T.L., Ling E., Haase C., Bresson D., Ursø B., von Herrath M.G. (2013). NKG2D blockade facilitates diabetes prevention by antigen-specific Tregs in a virus-induced model of diabetes. J. Autoimmun..

[104] Shalaby D., Saied M., Khater D., Abou Zeid A. (2017). The Expression of Activating Receptor Gene of Natural Killer Cells (KLRC3) in Patients with Type 1 Diabetes Mellitus (T1DM). Oman Med. J..

[105] Shi F.D., Wang H.B., Li H., Hong S., Taniguchi M., Link H., Van Kaer L., Ljunggren H.G. (2000). Natural killer cells determine the outcome of B cell mediated autoimmunity. Nat. Immunol..

[106] Homann D., Jahreis A., Wolfe T., Hughes A., Coon B., van Stipdonk M.J., Prilliman K.R., Schoenberger S.P., von Herrath M.G. (2002). CD40L blockade prevents autoimmune diabetes by induction of bitypic NK/DC regulatory cells. Immunity.

[107] Fraker C., Bayer A.L. (2016). The Expanding Role of Natural Killer Cells in Type 1 Diabetes and Immunotherapy. Curr. Diabetes Rep..

[108] Simoni Y., Diana J., Ghazarian L., Beaudoin L., Lehuen A. (2013). Therapeutic manipulation of natural killer (NK) T cells in autoimmunity: Are we close to reality?. Clin. Exp. Immunol..

[109] Marcenaro E., Carlomagno S., Pesce S., Moretta A., Sivori S. (2012). NK/DC crosstalk in anti-viral response. Adv. Exp. Med. Biol..

